# Copper Homeostasis in *Aspergillus fumigatus*: Opportunities for Therapeutic Development

**DOI:** 10.3389/fmicb.2019.00774

**Published:** 2019-04-12

**Authors:** Jinxing Song, Rongpeng Li, Jihong Jiang

**Affiliations:** The Key Laboratory of Biotechnology for Medicinal Plants of Jiangsu Province and School of Life Science, Jiangsu Normal University, Xuzhou, China

**Keywords:** *Aspergillus fumigatus*, copper homeostasis, antifungal therapeutic target, copper transporters, copper transcription factor

## Abstract

*Aspergillus fumigatus* can cause severe invasive aspergillosis in immunocompromised individuals. Copper, an essential but potentially toxic trace element for *A. fumigatus*, plays a critical role at the host-pathogen axis during infection. Accumulating evidence demonstrates that the host utilizes copper compartmentalization within macrophages to combat *A. fumigatus* infection. To survive under host-imposed copper toxicity, *A. fumigatus* has evolved sophisticated machinery to regulate copper homeostasis. Thus, targeting molecular pathways critical for copper homeostasis regulation provides an opportunity to improve therapeutic options for aspergillosis caused by *A. fumigatus*. In this review, we describe the copper homeostatic mechanisms by which *A. fumigatus* acquires and controls copper levels and explores the responses of the pathogen to alter copper levels in the host. Finally, we discuss the regulatory mechanisms of copper homeostasis that could be targeted for antifungal drug development.

## Introduction

*Aspergillus fumigatus* is one of the most prevalent airborne fungal pathogens, causing severe invasive aspergillosis in immunocompromised individuals (Brown et al., 2012a; Kwon-Chung and Sugui, 2013). Current therapies used to combat *A. fumigatus* that have limited efficacy. Only three major classes of antifungal drugs (polyenes, azoles, and echinocandins) are used in the clinic (Xie et al., 2014). However, the efficacy of these antifungal drugs is limited, and *A. fumigatus* and other pathogenic fungi are either intrinsically resistant or have developed resistance over time (Shapiro et al., 2011; Brown et al., 2012b; Robbins et al., 2017). Accordingly, new strategies and molecules to increase efficacy against pathogenic targets must be developed.

Copper is essential for many forms of eukaryotic life, but it can also be toxic due in part to its ability to generate reactive oxygen species (ROS) (Fridovich, 1983; Macomber and Imlay, 2009; Nevitt et al., 2012). This duality provides a promising measure for antifungal therapy development. Copper has historically been used to control fungal and bacterial growth in healthcare settings (Casey et al., 2010; Lazary et al., 2014). It has become apparent that hosts use copper to fight *A. fumigatus* infection, and *A. fumigatus* has also evolved some mechanisms to resist copper-mediated toxicity by regulating copper homeostasis (Kusuya et al., 2017; Wiemann et al., 2017; Cai et al., 2018; Park et al., 2018). Thus, copper plays an important role at the host–*A. fumigatus* axis, and a detailed understanding of copper homeostasis regulation in *A. fumigatus* can lead to new strategies for antifungal drug development. Therefore, it is worthwhile to provide an updated summary of *A. fumigatus* copper homeostasis, and highlight regulators of copper homeostasis as promising therapeutic targets for antifungal drug development.

## Core Components of the Copper Homeostasis System in *A. Fumigatus*

### Copper Transporters Are Involved in Copper Uptake

**Figure 1:**
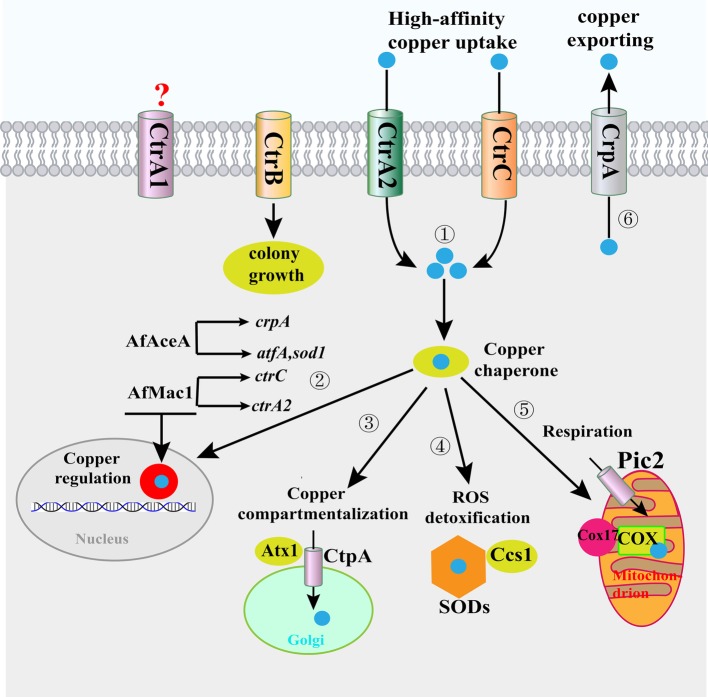
Copper homeostasis mechanisms in *A. fumigatus*. ① High-affinity copper transporters at the plasma membrane participate in copper (*blue spheres*) transport from the extracellular environment to the intracellular environment. ② In the cytoplasm, copper is bound by copper chaperones that facilitate the delivery of copper to the nucleus for copper homeostasis regulation; AfMac1 induces the *ctrC* and *ctrA2* genes to respond to low copper, and AfAceA induces the expression of the copper extrusion pump CrpA, as well as the ROS detoxification proteins AtfA and SOD1, in response to excess copper. ③ In the cytoplasm, copper is bound by copper chaperones that facilitate the delivery of copper to subcellular compartments (i.e., the Golgi complex) for storage or ④⑤ cytoplasmic and mitochondrial enzymes for functional activation. Parts 3, 4, and 5 are not proven in *A. fumigatus*, only in *S. cerevisiae*. ⑥ When the concentration of copper in the cytoplasm exceeds the homeostatic capacity, *A. fumigatus* employs efflux to detoxify excess copper. Abbreviations: SOD, superoxide dismutase; ROS, reactive oxygen species.

A copper uptake system at the fungal plasma membrane facilitates the import of copper across the plasma membrane and into the cytosol. Currently, much can be learned regarding the copper uptake system in *A. fumigatus* through examination of more well-studied systems in yeast (Smith et al., 2017). In the model yeast *Saccharomyces cerevisiae*, the copper uptake system consists of a low-affinity transporter protein (Fet4) and three high affinity transporter proteins, namely Ctr1, Ctr2, and Ctr3 (Dancis et al., 1994; Hassett et al., 2000; Pena et al., 2000; Rees and Thiele, 2007). Ctr1 and Ctr3, both of which are localized to the plasma membrane, transport copper into the cytosol. Meanwhile, Ctr2, which is localized to the vacuole membrane, pumps copper into the cytosol (Rees and Thiele, 2007). As Ctr family members are highly conserved across fungal species, *A. fumigatus* also encodes four putative Ctr family members designated as CtrA1, CtrA2, CtrB, and CtrC (Figure 1; Park et al., 2014), and each Ctr member has at least two transmembrane domains, with a characteristic Met-X (*n* = 1–5)-Met motif that is necessary for copper transport. Among the four transporters, both CtrA2 and CtrC have been identified as high-affinity copper transporters that are responsible for copper uptake from low-copper environments (Park et al., 2014). However, deletion of the *ctrA2* or *ctrC* gene did not affect the concentration of copper in the cells, which may be explained by the redundancy of these two genes. By contrast, double deletion of the *ctrA2* and *ctrC* genes dramatically reduced the intracellular copper content and resulted in growth and sporulation defects in copper-deficient medium (Park et al., 2014; Cai et al., 2017). Thus, CtrA2 and CtrC might play important roles in the regulation of copper homeostasis, growth, and sporulation of *A. fumigatus* in low-copper environments.

Although CtrA1 and CtrB exhibit homology with Ctr family members in *S. cerevisiae*, their exact function is unknown in *A. fumigatus*. Recent research demonstrated that deletion of the *ctrB* gene caused severe defects in hyphal growth in high- or low-copper environments, suggesting that CtrB affects the growth and morphology of *A. fumigatus* independently of copper homeostasis (Cai et al., 2017). Interestingly, the colony defects of the *ΔctrB* mutant could be suppressed by loss of the *ctrA2* or *ctrC* gene. Moreover, the high copper sensitivity phenotype of the *ΔctrA2* mutant could also be partially recovered by deletion of the *ctrB* gene (Cai et al., 2017). These results suggest that CtrA2, CtrC, and CtrB coordinately function to regulate hyphal growth and copper homeostasis, implying that Ctr transporters play a more complicated role in *A. fumigatus* than in yeast. To date, CtrA1 function in copper transport has not been revealed. Further studies are required to determine the nature of copper transport by CtrA1 in *A. fumigatus*.

### Copper Transporters Are Involved in the Efflux or Storage of Cytoplasmic Copper

It has been reported that P-type ATPases are important regulators of intracellular copper levels, and the recently identified P-type ATPase, CrpA, is the major determinant of copper resistance in *A. fumigatus* (Wiemann et al., 2017). In contrast to Ctr family importers, CrpA has been identified as a copper exporter (Figure 1), and *A. fumigatus* mainly uses CrpA to remove excess copper. CrpA mainly localizes to the cellular surface, and it is involved in the transport of copper from the intracellular milieu to the extracellular environment. Deletion of the *crpA* gene caused significant intracellular copper accumulation and a copper-sensitive growth phenotype (Wiemann et al., 2017). Another P-type ATPase copper transporter, Ccc2, is also involved in copper homeostasis in *S. cerevisiae* (Fu et al., 1995)*. A. fumigatus* also contains a potential homolog of *S. cerevisiae* Ccc2, designated CtpA (Figure 1). Similar to Ccc2 localization, CtpA is also localized on the Golgi complex in *A. fumigatus* (Upadhyay et al., 2013). Disruption of the *ctpA* gene leads to defective conidial pigmentation, which can be remediated by the addition of copper (Upadhyay et al., 2013). However, the role of CtpA as an exporter is yet to be clarified, and additional studies are required to determine the nature of copper transported by CtpA in *A. fumigatus*.

Cytochrome c oxidase (COX), the terminal enzyme of the electron transport chain in mitochondria, requires copper as a cofactor (Tsukihara et al., 1996). Accordingly, it is important to import copper into the mitochondrial matrix for the eventual assembly of COX. In *S. cerevisiae*, Pic2, a mitochondrial carrier family protein, mediates copper import into the mitochondrial matrix (Vest et al., 2013). Moreover, Pic2 is a conserved protein in *Aspergillus* species, but a role for Pic2 as a copper transporter in mitochondria has not been revealed in these species.

### Copper Chaperones Are Involved in Copper Trafficking

Because the copper buffering pool sequesters free copper in the cell, this copper then needs to be directed to the desired compartments. For this purpose, fungi have evolved highly specific and dedicated chaperones to direct copper trafficking. Currently, there are three copper transporting cytosolic chaperones (Atx1, Ccs1, and Cox17) in yeast (Smith et al., 2017). Atx1 is the copper chaperone responsible for delivering copper to the Golgi complex *via* Ccc2, whereas Ccs1 serves as the copper donor for superoxide dismutases (SODs). It has been demonstrated that Cox17 is responsible for transporting copper into mitochondria for the eventual assembly of COX. Although the chaperones Atx1, Ccs1, and Cox17 were only demonstrated in yeast, homologs of these three proteins are encoded in the genome of *A. fumigatus* (AFUB_008300, AFUB_025550, and AFUB_041410, respectively), but their function as chaperones is yet to be confirmed. Thus, further studies are required to determine their function in copper trafficking in *A. fumigatus*.

### Copper-Responsive Transcription Factors Are Involved in Copper Homeostasis

Copper homeostasis in fungi is primarily regulated at the level of transcription through copper-responsive transcription factors. Most fungi carry two copper-responsive transcription factors to respond to copper starvation or toxicity. Currently, much can be learned regarding the copper-responsive transcription factors in *A. fumigatus* through examination of more well-studied systems in yeast, and the copper status-specific transcription factors in *A. fumigatus* are similar to those found in *S. cerevisiae*.

In *S. cerevisiae*, copper starvation is sensed by the copper-responsive transcription factor Mac1, which activates Ctr1 and Ctr3 in response to low-copper environments (Jungmann et al., 1993; Smith et al., 2017). *A. fumigatus* also carries the *S. cerevisiae* Mac1 homolog AfMac1, the N-terminus of which contains the structure of conserved RGHR and GRP motifs for DNA binding. In addition, Cys-rich motifs are widely found at the C-terminus, which are involved in sensing copper (Cai et al., 2017; Kusuya et al., 2017). When *A. fumigatus* encounters low-copper concentrations, AfMac1 induces the *ctrC* and *ctrA2* genes. An additional EMSA result demonstrated that AfMac1 directly binds to a copper response element in the promoter regions of the *ctrA2* and *ctrC* genes with a defined consensus DNA motif (5′-TGTGCTCA-3′) (Park et al., 2017), which is strikingly similar to the Mac1-binding motif in *S. cerevisiae* (Jamison McDaniels et al., 1999; Keller et al., 2000), suggesting that the mechanism of Mac1-mediated copper homeostasis may be conserved across fungal species.

Despite the important roles of copper in fungal biology, excess copper is potentially toxic to fungal cells. Thus, many fungi also carry a second copper-responsive transcription factor to respond to excess copper. In *S. cerevisiae*, excess copper is sensed by the copper-responsive transcription factor Ace1, which activates expression of metallothionein (MT) genes as well as the copper-zinc SOD gene, in response to excess copper (Thiele, 1988; Gralla et al., 1991; Culotta et al., 1994). *A. fumigatus* also carries the *S. cerevisiae* Ace1 homolog AfAceA, and phylogenetic analyses revealed that most Ace1 homologs in fungi possess a conserved N-terminal signature including a zinc module and a copper-regulatory domain, suggesting that excess copper-responsive transcription factors might have a common mechanism of DNA binding in fungi (Cai et al., 2018). When *A. fumigatus* cells encounter high copper concentrations, AfAceA induces expression of the copper extrusion pump CrpA, as well as ROS detoxification proteins AtfA and SOD1 (Wiemann et al., 2017). Although AfAceA also activates expression of the metallothionein CrdA to respond to excess copper, *A. fumigatus* utilizes the copper export protein CrpA rather than the copper metallothionein CrdA as a primary copper resistance mechanism (Wiemann et al., 2017). Therefore, *A. fumigatus* may use a different mechanism to that of *S. cerevisiae* to manage copper toxicity, which mainly uses metallothioneins to detoxify excess copper. *S. cerevisiae* Ace1 recognizes a specific copper response element in target gene promoters with the consensus sequence 5′-TC(T)_4_6_GCTG-3′ (Furst et al., 1988; Huibregtse et al., 1989; Smith et al., 2017). However, we currently have a limited understanding of the AfAceA-binding motif in the target genes in *A. fumigatus*. Thus, further studies are needed to determine the AfAceA-binding motif in *A. fumigatus*, which will be one of the foci of future studies on the involvement of AfAceA in copper homeostasis regulation.

## The Copper Homeostasis System in Other Aspergilli

Recent studies have illustrated the copper homeostasis systems in other aspergilli. For example, there are two key genes involved in regulating copper homeostasis in *Aspergillus nidulans* (Antsotegi-Uskola et al., 2017). First, the *crpA* gene, encoding a P-type ATPase, is involved in the transport of copper from the intracellular milieu to the extracellular environment. In addition, CrpA expression is highly inducible and dynamic in response to prolonged copper exposure. Second, the *aceA* gene, encoding a transcription factor, is necessary for the copper-inducible expression of CrpA. Another *Aspergillus* species, *Aspergillus flavus*, possesses two copies of the copper exporter, CrpA and CrpB, which result in greater tolerance of copper in this species than in other aspergilli. Because of the redundant function of CrpA and CrpB proteins, only deletion of both genes resulted in extreme copper sensitivity. Similar to *A. fumigatus* and *A. nidulans*, *A. flavus* possess the same copper export machinery to prevent copper toxicity. In *A. flavus*, the Cu-fist binding transcription factor AceA senses high levels of copper, and induces the P-type ATPases CrpA and CrpB as a detoxification mechanism (Yang et al., 2018).

## Copper and *A. Fumigatus* Pathogenesis

### Copper-Based Strategies Deployed by the Host to Inhibit *A. fumigatus* Growth

ROS production and transition metal homeostasis (mainly iron and zinc) are the major strategies employed by host immune cells to kill *A. fumigatus* (Dagenais and Keller, 2009; Lanternier et al., 2013; Heinekamp et al., 2015; Clark et al., 2016; Kasahara et al., 2016). Currently, accumulating evidence suggests that hosts have also evolved an antimicrobial strategy based on copper poisoning of microbes enclosed in macrophages (Wiemann et al., 2017). Recent research demonstrated that copper and ROS within macrophages have a close relationship in the killing of *A. fumigatus* (Wiemann et al., 2017; Yang et al., 2018).

**Figure 2:**
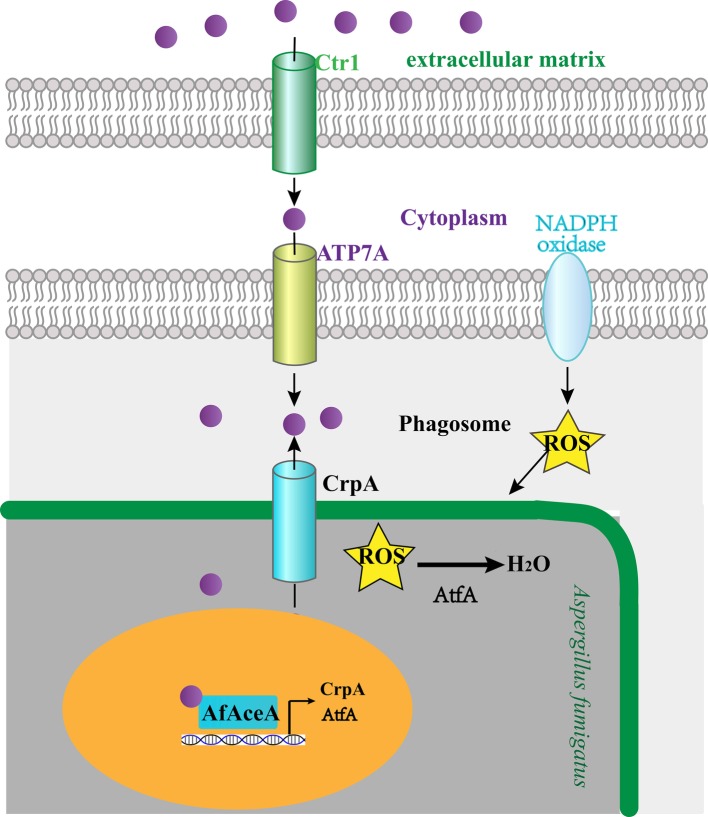
*A. fumigatus* response to host-imposed copper toxicity. Macrophages encountering *A. fumigatus* react by upregulating the host copper (*purple spheres*) transporter Ctr1 and aggregating ATP7A at the phagolysosomal membrane to facilitate copper uptake. *A. fumigatus* AfAceA utilizes the copper exporter CrpA and the ROS-responsive transcription factor AtfA to respond to host-imposed copper toxicity.

Although the precise mechanisms of copper mobilization in macrophages remain to be fully explored, some studies revealed that macrophages actively accumulate and compartmentalize copper by increasing the expression of the high-affinity copper transporter Ctr1 at the plasma membrane and translocating the P-type copper ATPase pump ATP7A to the phagolysosomal membrane (White et al., 2009; Achard et al., 2012; Ding et al., 2013; Garcia-Santamarina and Thiele, 2015). Because the host copper transport response to different microbial pathogens is generally conserved, macrophages encountering *A. fumigatus* spores also react by upregulating Ctr1 and aggregating ATP7A at the phagolysosomal membrane (Figure 2; Wiemann et al., 2017). When copper is transported into macrophages, it can potentiate redox potential and form highly-reactive hydroxyl radicals, thereby potentiating host-derived ROS toxicity (Figure 2; Thor et al., 1982; White and Clark, 1988). Therefore, host-derived ROS and copper responses could not be clearly separated in *A. fumigatus*, and copper mobilized by macrophages exerts its lethality by potentiating host-derived ROS toxicity.

### Copper Homeostasis and Virulence

Because host innate immune cells use copper compartmentalization as a means to exploit copper toxicity and enhance microbial killing, fungal pathogens possess highly orchestrated mechanisms to regulate copper homeostasis for preventing copper toxicity, thereby facilitating their survival. As mentioned above, *A. fumigatus* utilizes several protein classes to regulate copper homeostasis, including copper transporters (import and export) and copper-binding transcription factors (AfAceA and AfMac1), and these proteins play an important role in *A. fumigatus* virulence. Although the copper importers CtrA2 and CtrC failed to affect the mouse survival rate (Park et al., 2014), copper exporter CrpA is critical for *A. fumigatus* virulence as the *ΔcrpA* mutant showed significantly decreased virulence in an immunocompromised murine model of invasive aspergillosis (IA) (Wiemann et al., 2017). Because *crpA* was highly induced by copper in an AfAceA-dependent manner, AfAceA is also considered a virulence factor for *A. fumigatus*. In the non-neutropenic IA murine model, the *ΔAfAceA* mutant was significantly less virulent than the corresponding wild-type (Wiemann et al., 2017). Similar to AfAceA, another transcription factor, designated AfMac1, is also necessary for *A. fumigatus* virulence as deletion of *Afmac1* significantly reduced virulence (Cai et al., 2017; Park et al., 2017).

### *A. fumigatus* Response to Host-Imposed Copper Toxicity

To survive host-imposed copper toxicity, *A. fumigatus* has evolved some effective mechanisms to detoxify copper (Wiemann et al., 2017). *A. fumigatus* utilizes copper export as a primary copper resistance mechanism, and employs AfAceA to induce the copper exporter CrpA to prevent copper toxicity (Wiemann et al., 2017). In addition to copper export, *A. fumigatus* also activates AfAceA to induce the metallothionein CrdA as an intracellular copper buffer. Deletion of the *crdA* gene does not affect copper tolerance, whereas in *AfAceA* or *crpA* mutants, overexpression of *crdA* rescues copper sensitivity (Cai et al., 2018), thereby serving as a secondary mechanism of preventing host-imposed copper toxicity.

Macrophages generate ROS upon infection through the activity of the NADPH oxidase, which is an effective strategy for killing *A. fumigatus*. When copper is mobilized into macrophages, it can potentiate the redox potential and thereby oxidize lipids, nucleic acids, and proteins; these macromolecular insults have long been considered the primary basis for copper cytotoxicity (Garcia-Santamarina and Thiele, 2015). Taken together, copper mobilized by host cells partially exerts its lethality by potentiating host ROS toxicity. Thus, an efficient ROS detoxification system is critical for the survival of *A. fumigatus* within this hostile environment. *A. fumigatus* employs AfAceA to induce the ROS-responsive transcription factor AtfA, which is suggested to specifically govern spore ROS defense to cope with ROS stress within macrophages, and deletion of the *atfA* gene results in decreased survival in response to challenge with macrophages (Wiemann et al., 2017). Taken together, *A. fumigatus* AfAceA utilizes the copper exporter CrpA and the ROS-responsive transcription factor AtfA as the primary host countermeasures to host-imposed copper toxicity (Figure 2).

### Copper-Dependent Enzymes Involved in *A. fumigatus* Virulence

Melanin is a phenolic polymer and a well-known virulence factor (Langfelder et al., 2003; Nosanchuk and Casadevall, 2006). For example, melanin enhances fungal attachment to host tissues, absorbs host-generated ROS, and helps *A. fumigatus* evade host immune recognition (Thywissen et al., 2011; Bayry et al., 2014). Recent research has also demonstrated that *A. fumigatus* melanin can block LC3-associated phagocytosis to promote pathogenicity (Akoumianaki et al., 2016). Thus, *A. fumigatus* melanin is strongly associated with fungal virulence, and mutants defective in melanization are less virulent. In *A. fumigatus*, melanin synthesis requires the copper-dependent laccases Abr1 and Abr2, and deletion of either *abr1* or *abr2* significantly reduces melanin formation, making *A. fumigatus* spores more sensitive to destruction by macrophages (Pihet et al., 2009). As laccases require copper as their cofactor, dysregulation of copper homeostasis might affect laccase activity and/or expression (Upadhyay et al., 2013). Consistent with this conclusion, previous research found that the copper transporter CtpA is involved in copper homeostasis and that it plays an important role in supplying copper to laccases Abr1/2; thus, *ctpA* disruption leads to defective conidial pigmentation (Upadhyay et al., 2013).

In *A. fumigatus*, SODs are the most important family of enzymes for ROS detoxification, and the *A. fumigatus* genome encodes four putative SODs (*Af*SOD1–4). Among these four SODs, cytoplasmic Cu/ZnSOD1 and mitochondrial MnSOD2 play major roles in detoxifying ROS (Lambou et al., 2010). However, *A. fumigatus* SODs are not involved in fungal virulence, whereas SODs are required for virulence in *Cryptococcus neoformans* and *Candida albicans* (Cox et al., 2003; Narasipura et al., 2005). A reasonable explanation may be that *A. fumigatus* SODs are intracellular and therefore do not neutralize extracellular ROS produced by innate immune cells, which is different from the mechanism in *C. neoformans* and *C. albicans*. Accordingly, other ROS-scavenging pathways may play major roles in counteracting ROS in *A. fumigatus*. In conclusion, the copper-dependent enzyme laccase is closely related to *A. fumigatus* pathogenesis; however, Cu/ZnSODs are not apparently required for pathogenesis.

## Potential for Copper Homeostasis As a Novel Antifungal Therapeutic Target

Currently, the classic antifungal drugs used to treat fungal pathogens do not rapidly inhibit *A. fumigatus* growth, and hence, mortality rates remain unacceptably high (Xie et al., 2014). Thus, a major current challenge is developing new antifungal drugs that target *A. fumigatus*-specific metabolic pathways opposed to those targeted by the classic antifungal drugs. As previously described, host cells can inhibit microbial growth by intoxicating fungal pathogens with excess copper (Ding et al., 2013; Wiemann et al., 2017). Accordingly, one strategy could be therapeutically inhibiting the growth of fungal pathogens in the host, and the regulators of copper homeostasis have emerged as ideal targets for the development of novel antifungal therapies.

Because *A. fumigatus* has evolved efficient methods to counter copper-mediated killing though copper export (Wiemann et al., 2017), we propose that a therapeutic approach based on preventing copper extrusion would be deleterious for *A. fumigatus* cells. As the extrusion of copper is mainly mediated by the copper exporter CrpA in *A. fumigatus*, we speculate that CrpA could serve as a drug target, and any new compounds that interfere with the function of CrpA would predictably inhibit *A. fumigatus* growth within host tissues. In addition, CrpA has cysteine-rich copper-binding motifs (MBD) in its N-terminus, differing from the human homolog, and hence, any compound inhibiting the function of CrpA by targeting its N-terminus without affecting the human homolog would be theoretically valid. In *A. fumigatus*, the copper-responsive transcription factor AfAceA is the most important modulator of copper homeostasis and virulence, and *A. fumigatus* employs AfAceA to activate the copper exporter CrpA and ROS-degrading proteins to prevent copper toxicity. (Wiemann et al., 2017). Notably, the *ΔcrpA* mutant showed decreased virulence similar to that of the Δ*AfAceA* mutant in an immunocompromised murine model of IA, suggesting that the copper exporter plays a major role in *A. fumigatus* virulence. P-type ATPases are considered therapeutic targets due to their accessibility on cell membranes, coupled with the recent progress in specifically targeting P-type ATPases (Kirk, 2015; Turner, 2016). Inactivation of the P-type ATPase CrpA alone is sufficient to decrease virulence, and efforts to target CrpA may hold promise for future work. In summary, regulators of copper homeostasis have emerged as ideal targets for developing novel antifungal therapies. Future work should aim to discover and develop new drugs that specifically block the regulation of copper homeostasis.

### Copper Ionophores Could Potentially Serve As Antifungal Agents

Because regulators of copper homeostasis can be regarded as therapeutic targets, molecules interfering with copper homeostasis could be used as antifungal drugs. Copper ionophores can coordinate and shuttle copper from the extracellular environment to the intracellular milieu (Garcia-Santamarina and Thiele, 2015), thus, some studies found that copper ionophores can potentially serve as antifungal agents. For example, zinc pyrithione (ZPT), an antimicrobial agent widely used in antidandruff shampoos, can act as a copper ionophore (Chen and Hill, 2005; Reeder et al., 2011). However, ZPT is only useful in antidandruff shampoos due to toxic. The mechanism of action of ZPT as an antifungal agent has been investigated in *S. cerevisiae*, with data indicating that ZPT acts by dramatically increasing cellular copper concentrations to inhibit the growth of *S. cerevisiae* (Reeder et al., 2011). As observed in *S. cerevisiae*, ZPT also increases copper concentrations to inhibit the growth of *Malassezia globosa*, the most common fungus known to be associated with dandruff (Reeder et al., 2011).

Recently, boronic ester-masked 8-hydroxyquinoline derivative (QBP), which is a nontoxic protected form of the well-characterized copper ionophore 8-hydroxyquinoline (8HQ), has been used as an antifungal agent to inhibit the growth of *C. neoformans* in the lungs. The oxidative burst generated by activated macrophages mediates the conversion of QBP to 8HQ, which subsequently translocates copper into fungi within the phagolysosome, eliciting copper-dependent cytotoxicity (Festa et al., 2014). Therefore, the conditional activation of copper ionophores represents a promising approach to treating systemic fungal infections. Because 8HQ has broad-spectrum antimicrobial activity, QBP may potentially have a broad range of applications. Thus, future works are needed to apply this strategy to the treatment of *A. fumigatus* infection.

As mentioned above, copper ionophores are considered to be antifungal agents, mainly because they can increase the concentration of copper in cells. Thereby, compounds that inhibit the expression of copper detoxification genes and thus increase the intracellular copper concentration can also be considered as antifungal agents. AfAceA and CrpA are key proteins for copper detoxification and virulence in *A. fumigatus.* Thus, pharmacologic disruption of AfAceA or CrpA function would be predicted to induce fungal death due to the increased copper concentration in cells. Accordingly, AfAceA or CrpA could also be used as drug targets for the inhibitory effect of copper ionophores on fungal growth. Moreover, copper ionophores together with AfAceA or CrpA inhibitors could potentially magnify the copper-mediated toxicity.

## Future Directions and Conclusions

Because the expression of copper tolerance genes is critical for the survival and virulence of fungal pathogens, pharmacological disruption of the function of copper tolerance genes would be predicted to have the following beneficial effects: (1) **Induce fungal death**: the loss of copper tolerance genes would lead to toxic levels of copper that would be expected to have fungistatic and/or fungicidal effects on the organism. (2) **Enhance the activity of current antifungal drugs**: the clinical efficacy of current antifungal drugs may be compromised by intrinsic or acquired resistance. Combination antifungal therapy with agents of different mechanistic classes could promote fungal killing and clinical efficacy and provide an alternative to monotherapy regimens. This suggests that combining inhibitors of copper tolerance proteins with one or more current antifungals could increase efficacy, promote fungal killing, and prevent the emergence of drug resistance more effectively than monotherapy regimens. In particular, combination therapy might reduce the hepatotoxicity caused by long-term exposure to high doses of azoles drugs. Thus, the *AfAceA* and *crpA* copper tolerance genes may be potential drug targets, although deletion of these genes in *A. fumigatus* leads only to attenuated virulence, not avirulence. Taken together, the regulation of copper homeostasis provides promising therapeutic targets for combating *A. fumigatus* infection. Furthermore, by targeting copper homeostasis with a specific combination of two drugs, the possibility that the fungus will develop mutations that increase drug resistance is significantly lessened. Thus, future efforts should be directed to the discovery of new compounds that can specifically block the function of the proteins that regulate copper homeostasis. Because mammalian hosts can kill *A. fumigatus* by accumulating copper within the phagolysosome of macrophages, it is important to understand the underlying mechanisms of copper mobilization within macrophages and the corresponding fungal responses to excess copper. Biochemical approaches to manipulate copper homeostasis may provide promising strategies for the development of novel antifungal therapies.

## Author Contributions

JS conducted the literature study and wrote the manuscript. RL and JJ edited and revised the manuscript.

### Conflict of Interest Statement

The authors declare that the research was conducted in the absence of any commercial or financial relationships that could be construed as a potential conflict of interest.
